# Profiles of traditional healers and their healing practices in the Eastern Cape province of South Africa

**DOI:** 10.4102/sajpsychiatry.v24i0.1305

**Published:** 2018-09-20

**Authors:** Ntombizanele Menze, Alberta S.J. Van der Watt, Karis Moxley, Soraya Seedat

**Affiliations:** 1 Department of Psychiatry, Stellenbosch University, South Africa

## Abstract

**Background:**

Despite the widespread use of traditional healers in the management of mental health problems among South Africans, there is a knowledge gap in their practices that needs to be narrowed in order to develop a more collaborative and integrated mental health system. There is a need to better understand traditional practices from the perspective of the healers themselves and how these align with Western approaches.

**Aim:**

We specifically explored the journey towards becoming a traditional healer, the types of interventions and key practices in the management of mental disorders, and the extent to which traditional healers collaborate with conventional medical practitioners.

**Methods:**

This mixed-methods study involved 77 traditional healers who practice in the Eastern Cape province of South Africa. We administered semi-structured interviews to gather data on healer training, experiences and practices. The Patient Health Questionnaire (PHQ-9) was used to screen for depression. All interviews were conducted in isiXhosa at participants’ homes.

**Results:**

Most of the healers were female (80.5%) and only half (49%) had a traditional healing certificate. Healer training typically consisted of six key steps and was mostly facilitated by a non-family member or trainer, as directed by the ancestors. Most healers treated physical illnesses (86%) and called on their ancestors to assist with diagnoses (90%). Only 40% of healers treated mental illnesses. While some healers revealed tensions in working with Western practitioners, the majority were open to collaboration (71%).

**Conclusion:**

Traditional healers may have an important role to play in the development of culturally-relevant mental health care in South Africa. This study contributes to a greater understanding of what it means to be a traditional healer, and the types of treatment provided. The findings emphasise that conventional mental health practitioners need to make equal effort to collaborate, especially if we are to provide culturally-relevant mental health care in traditional South African settings.

